# Patient’s Education Before Mastectomy Influences the Rate of Reconstructive Surgery

**DOI:** 10.1007/s13187-016-0982-9

**Published:** 2016-01-21

**Authors:** R. Tarkowski, K. Szmigiel, A. Rubin, G. Borowiec, J. Szelachowska, W. Jagodziński, M. Bębenek

**Affiliations:** 1 Department of Oncology, Division of Surgical Oncology, Wroclaw Medical University, pl. Hirszfelda 12, 513-413 Wroclaw, Poland; 2 Students’ Scientific Society at the Department of Oncology, Wroclaw Medical University, pl. Hirszfelda 12, 513-413 Wroclaw, Poland; 3 Department of Oncology, Wroclaw Medical University, pl. Hirszfelda 12, 53-413 Wroclaw, Poland; 4 Analiza Badania Rozwój, Osiedle Stefana Batorego 11 lok 74, 60-687 Poznan, Poland; 5 1st Department of Surgical Oncology, Lower Silesian Comprehensive Cancer Center, pl. Hirszfelda 12, 53-413 Wroclaw, Poland

**Keywords:** Mastectomy, Breast reconstruction, Communication, Patient’s education

## Abstract

Breast reconstruction (BR) should be offered and discussed to each woman with breast cancer who planned for mastectomy, except the cases with severe comorbidities. However, the majority of these patients do not undergo reconstructive surgery. A 20-question survey was administered to a group of 50 women (age 29–83 years, median 53) treated with mastectomy. 22.4 % underwent reconstruction of the breast, 24.5 % declared an interest in BR in the future, 53.1 % were not interested in reconstructive surgery. 51.2 % obtained information concerning BR before surgery, 58.1 % after and 44.2 % both before and after mastectomy. 59.2 % were informed about reimbursement. Information given before surgery had a statistically significant impact on performing reconstruction or a declared interest in BR (*X* ^2^ = 4.950, *df* = 1, *p* < 0.05), as well as information about reimbursement (*X* ^2^ = 8.875, *df* = 1, *p* < 0.05). Age <55 years was another significant factor (*X* ^2^ = 13.522, *df* = 1, *p* < 0.05, C Pearson = 0.525). Level of education did not impact upon the choice (*p* > 0.05). The main reasons for the refusal were fear of complications (47.4 %), priority to recovery over aesthetic (36.8 %), age, defined by the patient as “advanced” (31.6 %), high level of acceptance of the body after amputation (31.6 %), fear of cancer recurrence (26.3 %) and fear of the pain and discomfort (15.8 %). Each patient who planned for mastectomy should obtain sufficient information regarding breast reconstruction. Exact information is of special benefit to women discouraged by imagined disadvantages of surgery. Patients’ education impacts the quality of life—not only before surgery but also lifelong after finishing the treatment.

## Introduction

Women with breast cancer are treated either with breast-conserving surgery (BCS) or mastectomy. Randomised trials show no benefit in terms of survival for more aggressive and mutilating mastectomy compared to BCS [1, 2]. In case of contraindications for BCS, there is access for breast reconstruction (BR)—either during a one-step, immediate procedure following ablation during the same surgical intervention (immediate breast reconstruction (IBR)) or for a delayed one, after finishing adjuvant treatment. Timing has no impact either on cosmetic effects [3] or on complication rates [4]. Resolution 2002/2279(INI) of the European Parliament about breast cancer treatment in the EU states: “wherever possible, breast reconstruction operations are performed using the patient’s own tissue and within the shortest possible time”. Similar recommendations exist across Europe, e.g. in the UK, where the UK National Institute for Health and Clinical Excellence (NICE) recommendations on *Early and Locally Advanced Breast Cancer*: *diagnosis and treatment* state clearly: “Discuss immediate breast reconstruction with all patients who are being advised to have mastectomy and offer it except where significant comorbidity or (the need of) adjuvant therapy may preclude this option. All apropriate breast reconstruction options should be proposed and discussed with the patients, irrespective of whether they are all available locally”. Recommendations of the Polish Society of Clinical Oncology (PTOK) indicate reconstruction as an integral element of surgical treatment and suggest that all patients planned for mastectomy should be informed about this option. Despite the NICE guidelines, the rate of breast reconstructions in the UK is low. Statistics performed in the Clinical Effectiveness Unit by the Royal College of Surgeons in England showed that only 16.5 % of mastectomy patients underwent immediate breast reconstruction in the 34-month period between April 2006 and February 2009 (7373 out of 44.837). In the subgroup of patients younger than 50 years, this percentage was higher, reaching 33 %. Reconstruction rates varied between 8 and 29 % among 30 cancer centres [5]. The National Mastectomy and Reconstruction Audit stated the proportion of women who underwent immediate breast reconstruction was 21 %, with a sharp rise comparing to 11 % from April 2005 to March 2006 [6]. BR rate across Europe does not exceed 20 % [7].

Reconstruction is the safe treatment option although there are no randomised controlled trials which support the effect of reconstructive surgery on survival [8]. Immediate BR does not lead to delay in the delivery of adjuvant systemic treatment and thus should not decrease survival rates [9]. It is widely assumed that BR positively impacts patients’ quality of life (QoL) through avoiding mutilation and decreasing psychosocial morbidity. However, there exists a proportion of mastectomy patients who cope well with their altered body image and proudly show their scars on photographs published by patient support organisations [10–12]. Furthermore, the results of a multicenter prospective study showed higher levels of satisfaction with women who had undergone mastectomy without BR than in other patients with reconstruction, either immediate or delayed [13]. In another study, cancer survivors with reconstructed breasts reported a negative impact of cancer on their sexual lives more often than the other patients (from BCS and mastectomy groups); however, they were also younger, more likely to be in relationships, better educated and more likely to undergo aggressive adjuvant chemotherapy. Perhaps it was not the choice of the kind of surgery, but all the aforementioned psychosocial and medical aspects of their characteristics impacted their quality of life [14].

Despite the proposal of BR, there still remains a group of women with breast cancer who prefer ablative surgery without any kind of reconstruction.

As surgical oncologists who offer BR and often encounter refusal or a lack of interest among our patients, we were curious about the reasons for this situation. Given the aforementioned data, the problem seems to be more complex than simply an insufficient service from BR providers. These were the reasons to look for factors making women with breast cancer neglect breast reconstruction.

## Aims

The aim of this study was to identify reasons for the low rate of breast reconstructions and to understand the motivations of women who refuse breast reconstruction after mastectomy.

## Materials and Methods

This cross-sectional study was conducted using a survey questionnaire on a sample of 50 patients with breast cancer (age 29–83 years, median 53) and operated on by means of mastectomy between 2000 and 2012 in Poland. Forty-eight percent had higher education, 48 % other than higher and 4 % did not indicate their education. 86.2 % of patients required adjuvant treatment: 50 % radiotherapy, 81 % chemotherapy and 51.7 % hormonal therapy.

**Fig. 1:**
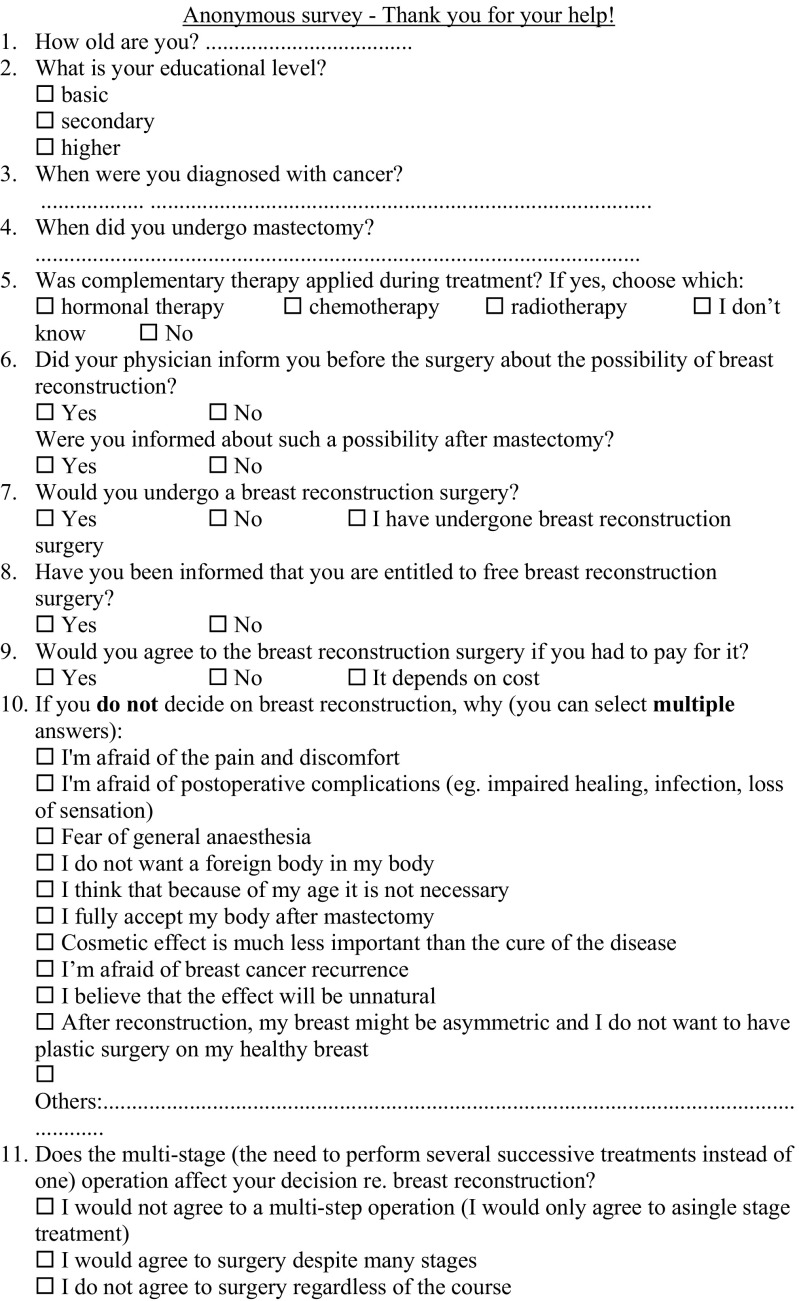

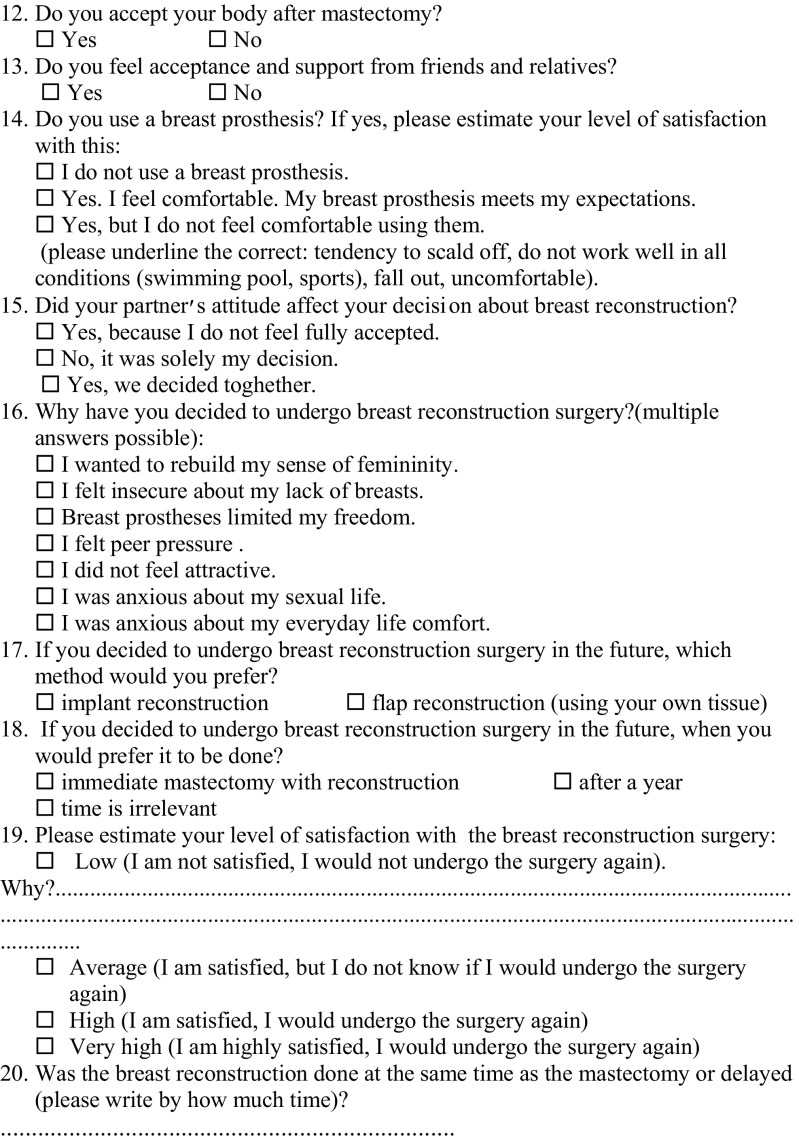
The survey

The data were collected in 2014. The survey consisted of 20 closed, open and semi-open questions—demographic data, and 16 questions regarding the subject of the study (Fig. 1) picked by the investigators as questions of interest or coming from patients and their group of support.

Questions were posed about the ability to obtain essential information on reconstructive surgery and financial issues (i.e. funding sources; Polish National Health System (NFZ) reimburses breast reconstruction, with some limitations, as we explained below); the level of self-acceptation and body image acceptance; support and acceptance from a partner and family and their influence on the decision to undergo breast reconstruction. The last part concerned quality of life with the use of an external breast prosthesis as a surrogate for BR. The survey was confidential.

In addition to an in-depth description of the statistical quantitative analysis, Pearson’s chi^2^ non-parametric tests were used to verify the existence of a relationship between the variables—including the key determinants for decisions about the breast reconstruction surgery.

## Results

22.4 % already underwent reconstruction of the breast, 24.5 % declared an interest in undergoing BR in future, whereas 53.1 % were not interested in reconstructive surgery. Thus, the overall proportion of the group who will have breasts reconstructed after treatment reaches about 47 %.

Only 51.2 % (*n* = 22) obtained information concerning breast reconstruction before surgery, usually from the operating surgeon, while 58.1 % were informed after surgery. 44.2 % were informed both before and after surgery. In contrast, 34.9 % did not hear about BR either before or after the operation (Table 1).

**Table 1:** Crosstable. Obtained the information concerning breast reconstruction before and after surgery

			Obtained the information concerning breast reconstruction after surgery		Total
			No	Yes	
Obtained the information concerning breast reconstruction before surgery	No	Frequency	15	6	21
		% within Total	34,9 %	14.0 %	48.8 %
	Yes	Frequency	3	19	22
		% within Total	7,0 %	44.2 %	51.2 %
Total		Frequency	18	25	43
		% within Total	41.9 %	58.1	100.0 %

Source: own calculations on the basis of research without missing values

Although the National Health System funds reconstruction, only 59.2 % were informed about the reimbursement.

Information about the possibility of BR obtained *before* surgery had a statistically significant impact on performing reconstruction or a declared interest in reconstructive surgery (*X* ^2^ = 4.950, *df* = 1, *p* > 0.05). Analysing the group of patients who underwent BR, we found that 72.7 % of them obtained such information before mastectomy. 57.1 % of them had already undergone reconstruction or had declared their desire to do so in the future. In contrast, only 27.3 % of women who were not informed about such a possibility before mastectomy underwent BR afterwards, despite information about reconstruction given after ablative surgery. The contingent C Pearson ratio for this group was 0.345 (*p* < 0.05)—this signifies a moderately strong relationship (Table 2).

**Table 2:** Crosstable. Information about the possibility of BR obtained before surgery and declared interest in reconstructive surgery (or reconstruction in the past)

			Declared interest in reconstructive surgery (or reconstruction in the past)		Total
			No	Yes	
Information about possibility of BR obtained before surgery	No	% with information about possibility of BR obtained before surgery	72.7 %	27.3 %	100.0 %
		% with declared interest in reconstructive surgery (or reconstruction in the past)	64.0 %	27.3 %	46.8 %
	Yes	% with information about possibility of BR obtained before surgery	36.0 %	64.0 %	100.0 %
		% with declared interest in reconstructive surgery (or reconstruction in the past)	36.0 %	72.7 %	53.2 %
Total		% with information about possibility of BR obtained before surgery	53,2 %	46.8 %	100.0 %
		% with declared interest in reconstructive surgery (or reconstruction in the past)	100,0 %	100.0 %	100.0 %

Source: own calculations on the basis of research without missing values

Information about BR given *after* mastectomy was not a significant discriminating factor for the patient population (*X* ^2^ = 1.648, *df* = 1, *p* > 0.05).

Information about reimbursement was a statistically significant factor influencing successful reconstruction (*X* ^2^ = 8.875, *df* = 1, *p* < 0.05). 82.6 % of patients who had already had BR and 71.4 % patients aged less than 55 years who had or were going to undergo BR were informed about the financial issues, in contrary to 17.4 who were not (C Pearson = 0472, *p* < 0.05). Twenty percent would choose BR when they should pay for surgery, while 44 % would refuse reconstruction without reimbursement; 36 % did not declare their opinion.

Level of education was not a significant factor in the choice, which was almost the same for all groups (*p* > 0.05). Age was a significant factor influenced decision to undergo BR with strong correlation: (*X* ^2^ = 13.522, *df* = 1, *p* < 0.05, C Pearson = 0.493). 71.4 % of women younger than 55 years had already undergone or were going to undergo reconstructive surgery, while only 14.3 % of older patients chose BR.

The main reasons for refusal were fear of postoperative complications (47.4 %), priority to recovery over aesthetic (36.8 %), age, defined by the patient as “advanced” (31.6 %), high level of acceptance of their body after amputation (31.6 %), fear of breast cancer recurrence (26.3 %), fear of the pain and discomfort (15.8 %), lack of foreign body (implant) acceptance (15.8 %), fear of assymetry (10.5 %) and an unnatural effect (5.3 %) (some of the patients indicated more than one answer, and this is the reason why the indications do not add up to 100 %).

## Discussion

Despite various guidelines stipulating the proposal of reconstruction in each case of planned mastectomy, the majority of patients do not undergo BR after mutilating surgery. Data published in the Annual Reports of the National Mastectomy and Breast Reconstruction (NMBR) Audit showed the different availability of breast reconstruction across the country [6]. There were several factors proposed to explain the regional variations, such as increased comorbidities in areas of social deprivation or different views of multidisciplinary teams concerning the timing of reconstruction and the low number of specialists trained in reconstruction techniques. There was another very important issue of a lack of information which was reported by 34 % of women and the insufficient time to make a decision—another 22 % [6, 15]. All these factors are common in our country, and this could explain the rarity of BR in Poland. In our study, almost half of the patients had not heard about BR from the surgeon, who would explain the treatment with its consequences during informed consent before the intervention. Probably, they did not obtain this information from a physician because he/she was not trained in reconstruction techniques. In Poland, these have traditionally been plastic surgeons who perform delayed reconstruction, usually working outside the medical centre where the mastectomy was performed. There are some, but not numerous, surgical oncologists who perform BR, either immediate or delayed, usually with the use of implants or pedicled flaps (i.e. LD—*latissimus dorsi* flap). Currently, certified Breast Units are a great rarity in Poland, although there are some surgeons dedicated to patients with breast cancer. Another cause of misinformation is the lack of specialised breast nurses. These professionals guide the patients through the therapeutic process and usually spend more time with them than a surgeon.

There are also some logistical problems which could explain the rarity of BR in Poland. Although *unilateral* reconstruction is reimbursed by the National Health Fund, NFZ does not fund either *bilateral* reconstruction or *symmetrisation* of the contralateral breast, when needed. Only unilateral surgical intervention is reimbursed during a single hospitalisation. Should we hospitalise patients with synchronic breast cancer or those who desire delayed bilateral reconstruction at least twice, performing surgery of the one side and repeating it afterwards for the contralateral one during another hospitalisation? There are also some difficulties concerning synchronic symmetrisation performed during the same intervention with BR. The majority of patients with breast cancer are more than 50 years old, when breast ptosis is not an uncommon feature. Present regulations are not only cost-inneffective for the system but also traumatic for the patients (as such, the most impressive counterargument for such approach) and cause a backlog of women waiting for treatment. This fear of repeated hospitalisations could explain the lower level of interest in BR among these patients. Although life-saving radical mastectomy eradicating cancer is an inherent part of the treatment, BR may not be thought worth the further inconveniences (as hospitalisation, pain, complications) for the vast majority of patients when viewed as an aesthetic surgery.

Another section of the respondents were not interested in reconstruction because of their high level of self-acceptance. These women simply do not feel they need BR and are accepting of mastectomy [10]. Perception about cancer changes through the first year after diagnosis and treatment, from fear of mutilation to acceptance of the consequences of therapy and the cessation of planning BR [14, 16]. This becomes even easier, when the scar and plane hemithorax are accepted by husband or partner and survivors obtain full support from their family, as seen in the present study. What seems paradoxal from the health point of view of the BR-providing surgeon is that the quality of life after mastectomy does not have to be lower than QoL after reconstruction. Harcourt explained this feature as the priority of cancer eradication at the time of diagnosis as a life-saving intervention over appearance-related issues [13]. For such patients, reconstruction should not be regarded as “the obvious next step” in a return to normal life and physicians should be cautious not to push patients towards BR and make them feel guilty, when they do not need that [10]. So although a BR proposal should be mandatory in each case of mastectomy, acceptance of the offer is highly variable. The younger patients accepted the offer of BR more often than the older ones. Although it may seem that older patients are often discouraged from breast reconstruction, we did not find this to be the case in our study.

## Conclusions

Each patient who planned for mastectomy should obtain sufficient information regarding breast reconstruction. Sufficient time should be spent on informing patients, both by surgeons and by specialised breast cancer nurses. The latter professionals should be activated as staff members capable to guide women with breast cancer through the therapy process. Educational sessions should be more than one short meeting before surgery. Sessions may be most beneficial for patients interested in BR, but discouraged by the imagined disadvantages of surgery. Information needs to be tailored to meet the expectations of the patient. While infrastructure problems such as lack of centres that provide BR and access to qualified physicians needs to be improved, it is not the only cause of low utilisation of reconstructive surgery. There is a large group of patients not interested in this surgery due to the high level of self-acceptance who should not be pushed towards reconstructive surgery. Patients’ education impacts the quality of life—not only before surgery but also lifelong after finishing the treatment.

## Limitations

The study was not a prospective one; thus, we could analyse the informations concerning what the patients perceive or remember what they were told prior to surgery. Another limitations are the number of patients and the year of diagnosis influencing issues of recall already mentioned above. There is also a historical effect on the availability of breast reconstructive surgery. BR was obtainable in the analysed period, although the availability could differ within time.

## Abbreviations

BCS: Breast-conserving surgery
BR: Breast reconstruction
BU: Breast unit
IBR: Immediate breast reconstruction
LD: *latissimus dorsi* flap
NFZ: National Health Fund (Poland)
NICE: National Institute for Health and Clinical Excellence
NMBR: National Mastectomy and Breast Reconstruction
QoL: Quality of life
PTOK: Polish Society of Clinical Oncology
